# Exploring Identity-By-Descent Segments and Putative Functions Using Different Foundation Parents in Maize

**DOI:** 10.1371/journal.pone.0168374

**Published:** 2016-12-20

**Authors:** Xun Wu, Yongxiang Li, Junjie Fu, Xin Li, Chunhui Li, Dengfeng Zhang, Yunsu Shi, Yanchun Song, Yu Li, Tianyu Wang

**Affiliations:** 1 Institute of Crop Science, Chinese Academy of Agricultural Sciences, Beijing, China; 2 Guizhou Institute of Upland Food Crops, Guiyang, Guizhou, China; Institute of Botany Chinese Academy of Sciences, CHINA

## Abstract

Maize foundation parents (FPs) play no-alternative roles in hybrid breeding because they were widely used in the development of new lines and hybrids. The combination of different identity-by-descent (IBD) segments and genes could account for the formation patterns of different FPs, and knowledge of these IBD regions would provide an extensive foundation for the development of new candidate FP lines in future maize breeding. In this paper, a panel of 304 elite lines derived from FPs, i.e., B73, 207, Mo17, and Huangzaosi (HZS), was collected and analyzed using 43,252 single nucleotide polymorphism (SNP) markers. Most IBD segments specific to particular FP groups were identified, including 116 IBD segments in B73, 105 in Mo17, 111 in 207, and 190 in HZS. In these regions, 423 quantitative trait nucleotides (QTNs) associated with 15 agronomic traits and 804 candidate genes were identified. Some known adaptation-related genes, e.g., *dwarf8* and *vgt1* in HZS, *zcn8* and *epc* in Mo17, and *ZmCCT* in 207, were validated as being tightly linked to particular IBD segments. In addition, numerous new candidate genes were also identified. For example, GRMZM2G154278 in HZS, which belongs to the cell cycle control family, was closely linked to a QTN of the ear height/plant height (EH/PH) trait; GRMZM2G051943 in 207, which encodes an endochitinase precursor (EP) chitinase, was closely linked to a QTN for kernel density; and GRMZM2G170586 in Mo17 was closely linked to a QTN for ear diameter. Complex correlations among these genes were also found. Many IBD segments and genes were included in the formation of FP lines, and complex regulatory networks exist among them. These results provide new insights on the genetic basis of complex traits and provide new candidate IBD regions or genes for the improvement of special traits in maize production.

## Introduction

Excellent maize (*Zea mays* L.) hybrids are important for providing sufficient food, feed and energy for the increasing needs of modern society. Maize hybrid breeding has undergone three stages: (1) double-cross hybrids using inbred lines derived from open-pollinated varieties, including landraces in the 1930s; (2) more productive single-cross hybrids in the late 1950s after the appearance of some heterotic groups, such as Iowa Stiff Stalk Synthetic (SS), Non-Stiff Stalk (NSS) and Iodent (IDT); and (3) superior commercial hybrids for breeding, which became increasingly privatized after the pre-1980s [1]. During the process of maize breeding, some important inbred lines were formed that contributed most to maize hybrid breeding [2]. Some of the most significant lines were widely used to develop new lines and excellent hybrids, such as B73, 207 (also known as PH207), Mo17 [1, 3], and Huangzaosi (HZS) [4]. Among the U.S. maize germplasm used from 1980 to 2004, most lines with Stiff Stalk (BSSS) background were derived from B73, and approximately 35% of Pioneer’s U.S. Central Corn Belt hybrids were from BSSS. Mo17 was widely used by all U.S. seed companies, and approximately 45% of Pioneer’s U.S. Central Corn Belt commercial hybrids have the pedigree of Mo17; 207 was an important parent in the development of 17 registered corn lines, and approximately 15% of Pioneer’s U.S. Central Corn Belt hybrids were derived from 207 [5]. HZS was derived from a Chinese landrace of Tangsipingtou and has been widely used in Chinese maize breeding programs. More than 40 elite lines and 80 hybrids were developed from HZS [4, 6]. These lines are defined as foundation parents (FPs) and play no-alternative roles in historical and current maize breeding programs. Understandably, some important genomic segments are often retained during the formation of FPs and their descendants under selection [7]. Such segments contain the same ancestral origins and are defined as identity-by-descent (IBD) regions [8], which have been demonstrated to be powerful in relatedness evaluation and mapping of genetic loci associated with phenotypic variations in animal and human research [9–11]. However, similar studies in plants, especially in maize, are scarce.

To evaluate the genomic and phenotypic characteristics of maize germplasm and help breeders develop new inbred lines and predict hybrid performance, many studies have focused on maize genetic differentiation [12, 13], pedigree information [14], and the genetic bases of complex traits using different panels with multiple sources, different molecular markers and differentiation patterns of maize germplasm from different ecological environments [15, 16]. For U.S. maize breeding, three main heterotic groups, i.e., Iowa Stiff Synthetic (SS), Non-Stiff Stalk (NSS) and Iodent (IDT), were formed in the late 1950s and constitute genetically distinct breeding pools today [3], with representative lines of B73, Mo17 and 207, respectively. Integrated with the pedigree information and high-throughput molecular makers, the current maize germplasm is further divided into Oh43, Lancaster, Oh07-Midland, Iodent, SS, commercial hybrid-derived groups, Argentine Maize Amargo background groups [3], A321, 207 [17], Tangsipingtou (TSPT), Ludahonggu (LDHG), Modified Reid, Lancaster, and P group [18, 19]. Common groups of B73, Mo17, 207, and HZS have been observed in different reports [4, 18, 20, 21]. These genetic differentiations resulted from genomic changes during maize artificial or natural selection [22], which caused some important phenotypic variations and provided opportunities for breeders to develop new inbred lines by assembling different genomic segments. Using linkage analysis [23, 24] and the genome-wide association study (GWAS) method [25], genetic loci associated with complex traits have been identified, and some important genes have been confirmed by positional cloning [26]. Using a next-generation sequencing strategy, Lai et al. identified 101 low sequence-diversity regions of the maize genome, and the results showed that only a few segments were retained during the formation of maize FPs [27]. Using the same strategy, Jiao and his colleagues analyzed the genomic differentiation among 278 temperate lines and identified extensive variable regions [28], but the relevant functions of these regions remain unknown. By combining genomic, transcriptomic and phenotypic analysis, the divergence between tropical and temperate lines and many genes involved in stress adaption were identified by Liu et al. [13], and the complex genetic networks during the improvement of maize adaptation were revealed. Most previous studies have addressed the relationships among maize accessions with different origins and clarified some important genetic networks of complex traits. However, there is no genome-wide account of the genomic basis of the formation of maize FP lines, and the IBD segments during the formation of maize FP descendants and relevant functions remain largely uncharacterized.

Here, we integrated one panel of 304 inbred lines, including the four maize FP lines of B73, Mo17, 207, and HZS and their corresponding descendants, and presented an in-depth analysis of genetic differentiation and genomic variation using a dataset of 43,252 single nucleotide polymorphism (SNP) markers. We performed a comprehensive analysis of IBD segments transmitted from these FPs to their descendants. Further, 180 representative lines of the 304 lines were selected to evaluate phenotypes based on the results of genotyping analysis. In total, 15 agronomic traits were investigated across multiple environments in three years, and a GWAS was conducted. The consistency between the GWAS results and quantitative trait loci (QTLs) mapping results was investigated to deduce the putative functions for each IBD segment. In addition, 9 representative lines with high membership (more than 0.75) and known pedigrees in each FP group were selected to extract RNA-seq data. Some candidate genes located within IBD segments were identified through genomic, transcriptomic and phenotypic variation analysis. The objectives of this study were to 1) identify specific phenotypes and IBD segments from a given FP and its descendants, 2) uncover the functions of IBD segments, 3) identify major genes involved in the formation of maize FP lines, 4) explore genomic patterns of maize FP formation, and 5) provide new insight for dissecting the genetic basis of complex traits.

## Materials and methods

### Plant materials

The four FP lines B73, Mo17, 207, and HZS and their relevant descendants were collected, including 304 lines with co-ancestor membership of greater than 0.5. These lines were divided into four FP groups according to the results of population structure analysis and pedigree information [29]. In total, 98 lines were appointed to the B73 group, 61 lines to HZS, 95 to 207, and 49 to Mo17. Detailed information is provided in S1 Table.

### Integration of the genotyping datasets

The newly collected 180 inbred lines were genotyped using the Maize SNP50 bead chip including 56,110 SNPs (http://support.illumina.com/array/array_kits/maizesnp50_dna_analysis_kit.html). When the maize seedlings were one month old, the leaves of five plants were sampled in bulk to extract genomic DNA according to the modified CTAB procedure [30]. These samples were genotyped at the Beijing Compass Biotechnology Company according to the Infinium® HD assay ultra-protocol guide. In addition, the SNP genotyping datasets of 124 other inbred lines were extracted from public datasets, including 400 accessions submitted by van Heerwaarden et al. [1], 280 accessions by Flint and his colleagues [31], and 367 elite lines by Wu et al. [4]. All genotypes from different panels were integrated according to the identical physical position based on the B73 reference genome (RefGen_v2) and marker names. Allele forms were transformed based on the complementary pair-wise base. The transcriptomic data for each gene in different organs were obtained from the public data bank of qTeller (http://qteller.com/qteller3/rna_data_sources.php). In addition, 36 inbred lines from a particular group of B73, Mo17, 207 and HZS were sampled according to the top ancestor memberships and known pedigree information, and the transcriptomic expression data of the genes in kernels at 15 days after pollination were extracted from the datasets published previously [15]. Finally, the integrated genotyping datasets, which included 43,252 SNPs, were successfully obtained for the 304 inbred lines according to the following SNP screening criteria: (1) the minor allele frequency (MAF) exceeded 0.05, (2) the missing rate was less than 0.2, and (3) the position of the marker was unambiguous on the physical map of the B73 reference genome (RefGen_v2).

### Analysis of population genetic structure

To evaluate population stratification and relatedness among entries, 304 lines were appointed to particular subpopulations using a model-based approach [32] and 5,000 SNPs with low missing rates and an even distribution across the genome. These lines were also grouped into different clusters based on the modified Euclidean genetic distance [33] using 43,252 SNPs, which was defined as follows: D = 1—identity by state similarity (IBS), with IBS being the probability that alleles derived at random from two individuals at identical loci are the same. For any two accessions, the probability of IBS was averaged over all non-missing loci. A cladogram was then constructed using the distance matrix described above based on the un-weighted pair group method with arithmetic mean algorithm (UPGMA) [34]. In addition, principal component analysis (PCA) of the 304 lines was performed [35] to visualize the population stratification. Model-based analysis was performed in STRUCTURE V2.3.3 [32], and the modified Euclidean genetic distance, construction of the cladogram based on UPGMA, and PCA analysis were conducted in TASSEL software 5.0 [36].

### Identification of identity-by-descent segments

According to the results of the population genetic structure analysis, one group containing a classical FP line and its descendants was denoted as the relevant FP group, which was named the representative FP line. For each group, genetic diversity (GD) was evaluated using 43,252 SNPs in PowerMarker V3.25 [37] and was defined as the probability that two alleles randomly chosen from the test sample were different [18]. SNPs with a GD of zero were defined as tag loci with no variation, as widely used in the identification of conserved regions [5, 7]. Then, comparisons between lines were performed by slide window in one given FP group, with a window size of 25 kilobases (kb) before and after each tag SNP. The average GD of the SNPs in one window was calculated to represent the variation of this segment. If one segment had a GD less than the given threshold, which was calculated as (average GD of one chromosome in a given subgroup) − 0.5 × SD (standard deviation for the chromosome in the given subgroup), it was defined as an IBD segment derived from the FP line. Then, the genetic frame diagram for each FP group was constructed based on the physical position of the B73 reference genome (RefGen_v2) using R software (https://www.r-project.org/).

### Phenotypic evaluation

By combining the results of principal component analysis (PCA), a total of 180 lines covering more than 99.72% of the genetic diversity of the original panel were sampled [37]. This panel was planted in 15 environments, including Beijing in 2011, 2012 and 2014 (spring sown); Gongzhuling in Jilin Province in 2014 (spring sown); Xinxiang in Henan Province in 2011, 2012 and 2014 (summer sown); Nanchong in Sichuan Province in 2011 and 2012 (spring sown); Tai'an in Shandong Province in 2011 and 2012 (summer sown); Haerbin in Heilongjiang Province in 2011 and 2012 (spring sown); and Xinjiang in 2011 (spring sown). At each location, the lines were planted based on a randomized experimental design. These experimental locations belong to Chinese Academy of Agricultural Sciences, so no permission was needed. The field studies did not involve any endangered or protected species. At each location, the lines were planted based on a randomized experimental design. Plants were sown in single rows, with a row length of 4 m (15 plants per row) and a row width of 0.6 m, with two replicates, and with a plant density of 52,400 plants per hectare. Fifteen main agronomic traits were investigated. Days to tasseling (DTT), days to silking (DTS) and days to pollen-shedding (anthesis) (DTP) were recorded when 50% of the plants exhibited the corresponding traits. Then, the anthesis and silking interval (ASI) was calculated according to the formula ASI = DTS—DTP. On the 15th day after pollination, five continuous plants starting from the third plant were selected in one row to measure the ear height (EH) and plant height (PH), and EH/PH was calculated. After harvest, five representative ears from the center of each plot were sampled to investigate yield-related traits: ear length (EL) and ear diameter (ED) were measured in cm with a ruler; ear row number (ER) and kernel number per row (KNPR) were counted; 100-kernel weight (KWE) and 100-kernel volume (KV) were determined three times using an electronic balance with a weight range between 0.001 g and 1000 g, calculating the average value of three samples; and 10-kernel length (10KL) and 10-kernel width (10KW) in cm were measured by selecting 10 kernels from the center of each ear, followed by calculation of 10KL/10KW. Finally, kernel density (KD) was calculated as KWE divided by KV. The 15 traits were classified into four groups: plant architecture-related traits (PT-), which include EH, PH and EH/PH; flowering time-related traits (FT-), which include DTP, DTS, DTT and ASI; kernel-related traits (KRT-), which include KL, KW, KD, KV and KNPR; and ear-related traits (ET-), which include ED, EL and ER.

### Phenotypic data analysis

ANOVA was performed using the PROC GLM model. Pearson correlation analysis of each trait across different environments was calculated using the PROC CORR model. Then, the best linear unbiased predictor (BLUP) calculation for each trait was implemented using a PROC MIXED model, with genotype, location, genotype by location, and replications as random effects [38]. All analyses described above were completed using SAS (Release 9.3; SAS Institute, Cary, NC, USA).

### Genome-wide association study (GWAS)

The BLUP values of all agronomic traits for each line across different environments and 43,252 SNPs were used to perform a phenotype-genotype GWAS, which was implemented in the R package GAPIT [39] using the mixed linear model (MLM) with population structure and pair-kinship treated as covariates [40]. The significance cutoff value was defined as 0.05 divided by the number of markers. Quantitative trait nucleotides (QTNs) were selected for further analysis when the *p* values of the SNPs were less than the cutoff value.

### Comparison of genetic loci supported by IBD-based analysis, linkage mapping and GWAS

QTLs associated with the 15 agronomic traits were collected from public data and the literature (http://www.maizegdb.org/). The physical positions of each IBD segment, QTL, and QTN were queried against the B73 reference genome (RefGen_v2). On this basis, consistency analysis was conducted by projecting IBD segments, QTLs, and QTNs onto the identical genetic frame diagram. IBD segments containing both QTLs and QTNs were treated as important candidate genetic regions and were analyzed in depth.

### Candidate genes in important IBD regions

For each important IBD region, genes predicted by the Filtered Gene Set model [41] and located within 25 kb from the tag SNP were treated as tightly linked candidate genes based on the average LD of 30 kb, in contrast to the range of 20–50 kb used for a diverse panel in our previous research [38], and were screened to conduct further analysis. Gene function annotations and syntenic alignment between maize and the other model plants were conducted according to the method described previously [42]. In addition, we selected nine representative lines in each FP group and investigated their expression level in kernels at 15 days after pollination based on data extracted from previous reports[15]. The expression of each gene was evaluated by the fragments per kilobase of exon per million fragments mapped (FPKM). Based on the normalization of the expression data defined as log_2_(FPKM) [15], ANOVA between different FP groups was performed using the PROC GLM model, and Pearson correlation analysis was carried out using the PROC CORR model. These analyses were conducted using SAS software (Release 9.3; SAS Institute, Cary, NC). Gene interaction networks based on the expression data in 27 maize organs were visualized using Cytoscape V.3.2.1 [43]. Integrating these results together, genes tightly linked to tag SNPs, located within QTL and IBD regions and exhibiting particular expression trends in a given FP group were defined as important candidate genes for the formation of the FP lines.

## Results

### Analysis of population structure

**Fig 1:**
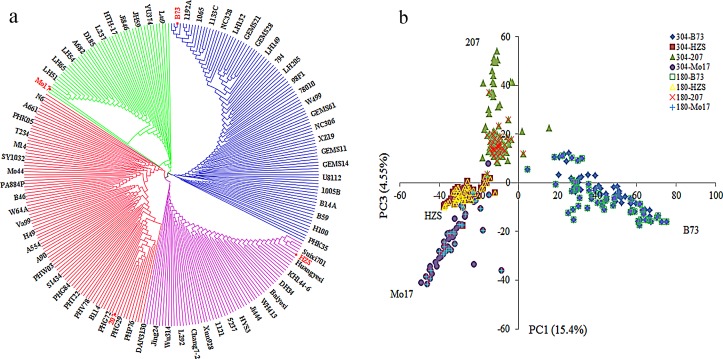
Genetic structure described by clustering and principal component (PC) analysis. “a” presents the cladogram constructed using the un-weighted pair group method with arithmetic mean algorithm (UPGMA) based on the modified Euclidean genetic distance. “b” shows the genetic structure described by PC1 and PC3 obtained from PCA on the 304-line panel and the 180-line panel, with 304- and 180- in the picture legend. The numbers inside the brackets show the proportion of the total variance for each PC.

The 304 lines were clustered into four groups (Fig 1A) corresponding to the FP groups of B73, 207, HZS, and Mo17. The B73, 207, HZS and Mo17 groups contained 99, 95, 61 and 49 lines, respectively (S1 Table). PCA showed that the first three PCs could explain 28% of the variance, and the four groups were divided using PC1 and PC3, with B73, 207, HZS and Mo17 distributed in the outer vertex of each triangle (Fig 1B), in good agreement with the results of both the model-based population structure and clustering analyses. One subset of 180 lines was sampled from the 304 lines based on the results of the population structure analysis. The results indicated that this new sub-panel covered 99.72% of the genetic diversity of the original population (Fig 1A and 1B).

### Phenotypic variations

Based on the data of the 15 main agronomic traits in the 180 lines, specific variation characteristics were found in different FP groups (S1A, S1B, S1C, S1D and S1E Fig). For example, the lines in the 207 group exhibited the shortest flowering time, including the smallest values of DTP, DTT and DTS. In addition, the smallest EH was observed in the 207 group. The B73 group exhibited the largest values of PH and 10KL/10KW and the smallest values of KV. The HZS group exhibited the largest values of EH/PH, ED, 10KW and KV and the smallest values of 10KL/10KW and KD. The Mo17 group exhibited the largest values of EL, KV and KNPR and the smallest values of ER. These abundant phenotypic variations facilitated the exploration of the functions of IBD segments during the formation of the FPs.

### Genome-wide association study of agronomic traits

The values of BLUPs for the 15 agronomic traits of the 180 lines across 15 environments were used in phenotype-genotype association mapping. The major results are shown in S2A, S2B, S2C and S2D Fig and S2 Table. In total, 423 QTNs were identified as being significantly associated with the 15 agronomic traits, of which 183 QTNs were significantly associated with plant architecture-related traits (PT), including 25 EH-, 35 PH- and 123 EH/PH-QTNs. Sixty-eight QTNs were significantly associated with flowering time-related traits (FT), including 13 DTP-, 10 DTS-, 37 DTT- and 8 ASI-QTNs. One hundred and twelve QTNs were significantly associated with kernel-related traits (KRT), including 4 KL-, 6 KW-, 29 KD-, 7 KV- and 66 KNPR-QTNs. Fifty-nine QTNs were significantly associated with ear-related traits (ET), including 17 ED-, 24 EL- and 18 ER-QTNs. Among these QTNs, 20 were significantly associated with multiple traits, including two QTNs, PZE-107101504 and PZE-109085452, that were significantly associated with EH, EH/PH, DTP and DTT. In addition, eight QTNs were significantly associated with three traits, including SYNGENTA17044, which was tightly linked to the gene *vgt1* on chromosome 8 and was significantly associated with DTP, DTS and DTT.

### Identity-by-descent (IBD) segments in different FP groups and putative functions

**Fig 2:**
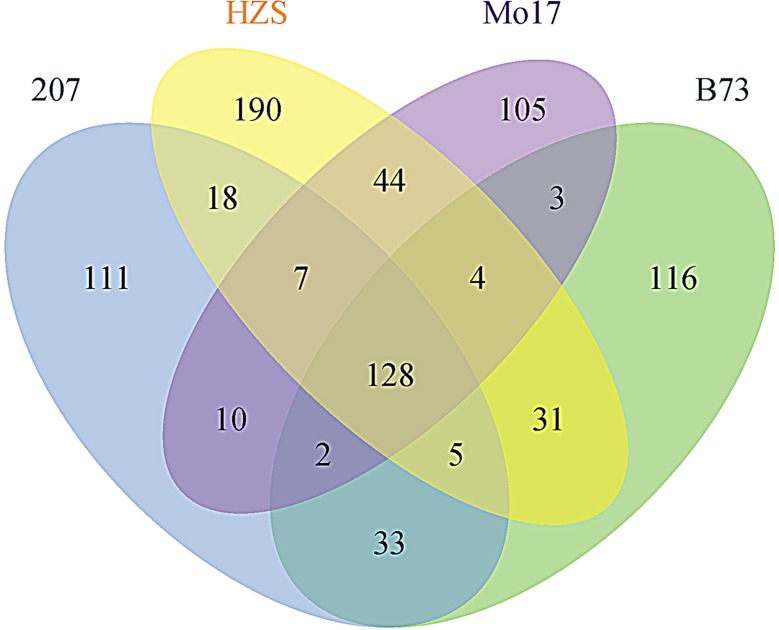
Comparison of identity-by-descent (IBD) regions between foundation parent groups.

**Fig 3:**
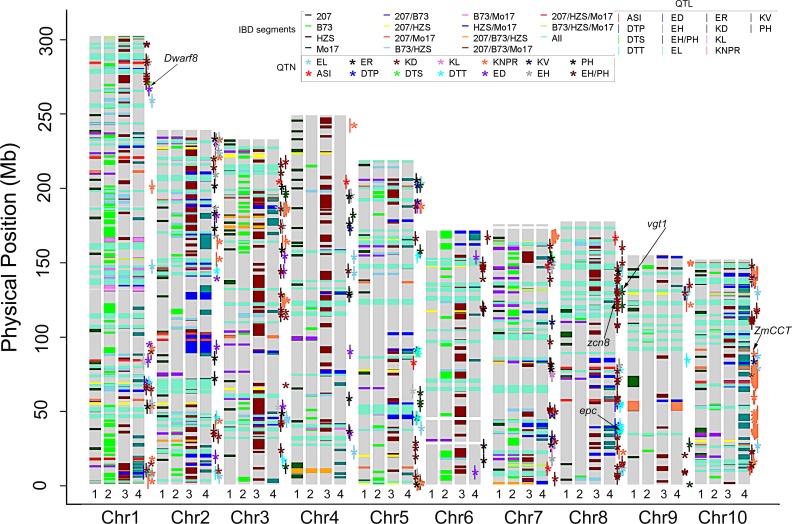
Genetic frame diagram of the identity-by-descent (IBD) regions for different foundation parent (FP) groups. The numbers “1”, “2”, “3”, and “4” below each histogram represent 207, B73, HZS, and Mo17, respectively. Transverse lines (“—”) of different colors represent IBD regions for different FP groups. Asterisks (*) and vertical lines (“|”) of different colors represent QTNs and QTLs significantly associated with different agronomic traits, respectively.

The results of the IBD segments are shown in Fig 2 and Fig 3. In total, 322 segments showed IBD between lines in the B73 group, as did 314 segments in the 207 group, 427 segments in the HZS group, and 303 segments in the Mo17 group. Pair-wise comparison showed that 168, 158 and 147 segments were common in 207 *vs*. B73, 207 *vs*. HZS and 207 *vs*. Mo17, respectively; 168 and 137 segments were common in B73 *vs*. HZS and B73 *vs*. Mo17, respectively; and 183 segments were common in HZS *vs*. Mo17. Triple comparison showed that 133 segments were common among B73, 207 and HZS; 130 among B73, 207 and Mo17; 135 among HZS, 207 and Mo17; and 132 among B73, HZS and Mo17. Quadruple comparison showed that 128 segments were common among these four groups. By contrast, 116 IBD segments were specific to the B73 group, 111 to 207, 190 to HZS, and 105 to Mo17 (Fig 2).

To explore the functions of the IBD segments, all IBD regions were compared with the QTNs from the GWAS and the QTLs from linkage mapping (Fig 3, S2 Table). Some genetic loci related to the 15 main agronomic traits were located within the IBD regions of the FP groups. A total of 423 QTNs located within the IBD regions specifically existed in a particular FP group and were significantly associated with FT-related traits, PT-related traits, KRT-related traits and ET-related traits, and these QTNs overlapped with relevant QTLs supported by linkage mapping. Eighty-two of these genetic loci showed clustered distributions across the genome. For example, three QTNs, PZE-101216827, PZE-101220442 and PZE-101222852, were associated with ED, DTS and EH/PH, respectively, and were tightly linked and located within the ear-related QTL of *ers25-IDP4143*, the flowering time-related QTL of *m0136*, and the plant height-related QTL of *qplht75*, respectively (S2 Table). Comparison analysis showed that these genetic loci were located within an IBD region specific to the HZS group. The IBD region specific to the B73 group on chromosome 7 contained one QTN/QTL cluster, including eight QTNs and four QTLs associated with FT-related traits, PT-related traits and KRT-related traits. The IBD region specific to the 207 group on chromosome 8 contained three EH/PH-related QTNs and one PH-related QTL. The IBD region specific to the Mo17 group on chromosome 10 contained 10 QTNs and two QTLs associated with KNPR and ear-related traits.

### Transcriptomic expression of genes in identity-by-descent (IBD) segments

**Fig 4:**
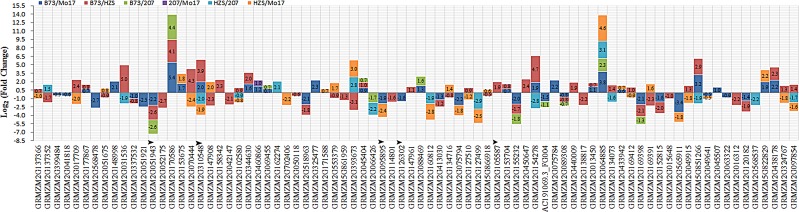
Expression of genes in kernels. The arrows indicate genes commonly expressed in kernels 15 days after pollination. The histogram shows the change in the expression level between different FP groups. The number in each histogram is the log_2_(fold change), and a minus sign before a number indicates the direction of change.

We compared the gene expression levels in different FP group pairs using transcriptomic data of kernels at 15 days after pollination. In total, 80 genes showed significantly different expression levels, with log_2_(fold change) varying from -3.45 to 5.39, where a minus sign indicates a downward change (Fig 4, S4 Table). For the B73 group, 28 genes were up-expressed, and 24 genes were down-expressed. For instance, GRMZM2G031536, which was tightly linked to one QTN of kernel density and was located within specific IBD regions of the B73, HZS and 207 groups, showed down-expression compared with the HZS/B73 pair. For the HZS group, eight genes were up-expressed, and 17 genes were down-expressed. For example, GRMZM2G154278, which belongs to the cell cycle control family, was closely linked to the QTN of the EH/PH trait and was located within one IBD region specific to the HZS group that was down-expressed in the HZS group compared with the HZS/B73 and HZS/207 pairs (Fig 4). or the 207 group, two genes were up-expressed, and one gene was down-expressed. For instance, GRMZM2G051943, which encodes an endochitinase precursor (EP) chitinase, was co-expressed with 21 other genes and was located within one IBD region of the 207 group that was tightly linked with one QTN for kernel density. For the Mo17 group, five genes were up-expressed, and six genes were down-expressed. For example, GRMZM2G170586 showed higher fold change and was located within an IBD region of the Mo17 group (Fig 3) and tightly linked with one QTN of ear diameter. However, no syntenic genes were found when blasting against the public databases of *Arabidopsis* and rice (S3 Table).

## Discussion

### IBD regions play no-alternative roles during maize foundation parental line formation

Many of the new commercial lines used today were developed from only a few FP lines. For instance, B73 was a pivotal line in the development of Stiff Stalk accessions and incurred the most patent hits of any inbred line, either public or proprietary. Mo17 is widely used during new line development within Holden’s Foundation Seeds. LH51 was derived from Mo17, with a similarity of approximately 97%, and is the predominant lineage of Lancaster germplasm. These two lines were used by nearly all seed companies in the USA. Additionally, 207 was the FP in the development of Iodent background inbred lines, and more than 17 registered corn lines have been derived from this line during the U.S. maize breeding process [3]. In Chinese maize breeding, more than 40 elite lines and 80 hybrids have been derived from HZS [6]. Liu et al. [7] used three FPs of Dan340, HZS, and Mo17 and 23 relevant derivatives to explore the IBD segments in different FP lines and their descendants and identified 26, 25, and 23 IBD regions, respectively. In this paper, among the FP lines of B73, Mo17, 207, and HZS and their relevant descendants, 128 conservative genetic regions were determined to be identical (Fig 2), which may account for some common phenotypes that were improved during the maize breeding process due to the common corn breeding objectives of high yield, good quality, wide adaptation and strong stress tolerance [12]. These objectives led to the retention of some commonly important genetic regions during artificial selection and improvement. Most importantly, many specific genetic regions were also identified in this study, including 116 specific to B73, 105 to Mo17, 190 to HZS, and 111 to 207. The assembly of special IBD regions included in the genome of a given group accounted for the largest PH in B73, the largest 10KW and EH/PH in HZS, the longest EL and smallest ER in Mo17, and the shortest DTT and DTP in 207, providing a good opportunity for discovering relevant genetic loci for determining the genetic basis of complex traits. In addition, several known genes related to important agronomic traits were located within specific IBD regions. For example, *vgt1*, which is associated with flowering time [44], was located within the IBD region of HZS, which was tightly linked to DTT-, DTP-, and DTS-related QTNs and QTLs. In addition, a total of 56 FT-related traits, PT-related traits and yield-related candidate genes were also located within the specific IBD regions of B73, HZS, Mo17 and 207 (Fig 3, S2 Table). These IBD regions may have played major roles during the formation of FP lines and account for special phenotypic variations.

### Candidate genes in IBD regions

In this paper, five known genes associated with FT-related traits were also identified using a GWAS and linkage analysis, including *dwarf8* [45], *vgt1* [44], *zcn8* [46], *ZmCCT* [47] and *epc* [48]. These genes are located within the specific IBD regions of HZS (Fig 3), which may explain why HZS and its descendants are widely used in the Huanghuaihai summer-maize region [49], where maize-wheat rotation is performed in the same field. A shorter growth period of maize would reduce the competition between maize and the next crop. Therefore, hybrids with shorter FT-related traits are popular in these regions. One breeding objective was to reduce the maize growth period in the Huanghuaihai summer-maize region; thus, many special loci related to shorter FT-traits were selected and fixed during the improvement, followed by the formation of special IBD regions in the particular FP and its descendants because HZS is a classic FP line with shorter FT-related traits [49]. Therefore, many loci related to shorter FT-related traits were retained in the HZS-derived lines. More interestingly, new candidate genes were also identified that were located within the specific IBD regions of B73, HZS, Mo17 and 207. For instance, GRMZM2G154278, which belongs to the cell cycle control family, was closely linked to a QTN of the EH/PH trait and was located within the IBD region specific to the HZS group, consistent with the highest value of EH/PH for the HZS group lines. GRMZM2G154278 was down-expressed in the HZS group compared with the B73 and 207 groups (Fig 4). For the 207 group, GRMZM2G051943, which encodes an endochitinase precursor (EP) chitinase, was located within one specific IBD region of the group, tightly linked to a QTN of kernel density and co-expressed with 21 other genes. Syntenic alignment showed that this gene is homologous to a homolog of carrot EP3-3 chitinase in *Arabidopsis*, which functions in somatic embryo formation [50]. For the Mo17 group, GRMZM2G170586, which was located within a specific IBD region, exhibited a higher fold change of gene expression and was tightly linked to one QTN of ear diameter (ED), consistent with the smallest value of ED in the group. These genes are important candidate genes for specific phenotypic variations in the given FP groups, which would provide reliable information for dissecting the genetic basis of complex traits.

## Conclusions

In this paper, we identified special IBD segments in particular FP groups that contained 82 QTN/QTL clusters located within different specific IBD regions, including known genes associated with FT-related traits and PT-related traits. These IBD regions may have played major roles during the formation of FP lines and account for special phenotypic variations. The results provide a good opportunity for discovering relevant genetic loci for determining the genetic basis of complex traits when integrating different FP groups together.

## Supporting Information

S1 Fig Box plot of agronomic traits in the four foundation parent groups. The letter on each box shows multiple testing, and a significant difference at 0.05 between two groups is indicated with different letters. “a” shows ear-related traits, including ear length (EL), ear diameter (ED), ear row number (ER), and kernel number per row (KNPR). “b” shows flowering time-related traits, including DTP, DTS, DTT, and ASI. “c” shows kernel shape-related traits, including 10-kernel length (10KL), 10-kernel width (10KW), 10KL/10KW, and 10-kernel thickness (10KT). “d” shows plant architecture-related traits, including plant height (PH), ear height (EH), and EH/PH. “e” shows kernel weight-related traits, including 100-kernel weight (KWE), 100-kernel volume (KV), and kernel density (KD).
(TIF)

S2 Fig Manhattan plot of the GWAS results. Red and gray lines are defined as 0.05 and 0.01 divided by the SNP number of 43,252, respectively. “a” shows ear-related traits, including ear length (EL), ear diameter (ED), ear row number (ER), and kernel number per row (KNPR). “b” shows flowering time-related traits, including DTP, DTS, DTT, and ASI. “c” shows yield-related traits, including 10-kernel length (10KL), 10-kernel width (10KW), kernel density (KD) and 100-kernel volume (KV).
(TIF)

S1 Table Summary information of the pedigrees and co-memberships of the 304 lines (XLSX)

S2 Table GWAS results of 15 agronomic traits and relevant linked QTL/genes. EH, PH, EL, ED, KD, KNPR, DTT, DTP, DTS, ASI, KV, 10KW, 10KL, and ER are the abbreviations for ear height, plant height, ear length, ear diameter, kernel density, kernel number per row, days to tasseling, days to pollination, days to silking, interval between silking and anthesis, 100-kernel volume, 10-kernel width, 10-kernel length and ear row number, respectively.
(XLSX)

S3 Table Candidate genes located within 50 kb of the peak SNPs. (XLSX)

S4 Table Comparison of gene expression between the foundation parent groups in kernels at 15 days after pollination. FPKM is the abbreviation for fragments per kilobase of exon per million fragments mapped.
(XLSX)

## References

[1] van Heerwaarden J, Hufford MB, Ross-Ibarra J. Historical genomics of north American maize. Proceedings of the National Academy of Sciences of the United States of America. 2012; 109(31):12420–12425. 10.1073/pnas.1209275109 22802642 PMC3412004

[2] Smith S. Pedigree background changes in US hybrid maize between 1980 and 2004. Crop Sci. 2007; 47(5):1914–1926.

[3] Mikel MA, Dudley JW. Evolution of north American dent corn from public to proprietary germplasm. Crop Sci. 2006; 46(3):1193–1205.

[4] Wu X, Li Y, Shi Y, Song Y, Wang T, Huang Y, et al Fine genetic characterization of elite maize germplasm using high-throughput SNP genotyping. Theor Appl Genet. 2014; 127(3):621–631. 10.1007/s00122-013-2246-y 24343198

[5] Smith JSC, Duvick DN, Smith OS, Cooper M, Feng LZ. Changes in pedigree backgrounds of pioneer brand maize hybrids widely grown from 1930 to 1999. Crop Sci. 2004; 44(6):1935–1946.

[6] Li Y, Wang TY. Germplasm base of maize breeding in China and formation of foundation parents. Journal Maize Sci. 2010; 18:1–8 (in Chinese).

[7] Liu CL, Hao ZF, Zhang DG, Xie CX, Li MS, Zhang XC, et al Genetic properties of 240 maize inbred lines and identity-by-descent segments revealed by high-density SNP markers. Mol Breeding. 2015; 35:146

[8] Browning SR, Browning BL. High-resolution detection of identity by descent in unrelated individuals. Am J Hum Genet. 2010; 86(4):526–539. 10.1016/j.ajhg.2010.02.021 20303063 PMC2850444

[9] Stevens EL, Heckenberg G, Roberson EDO, Baugher JD, Downey TJ, Pevsner J. Inference of relationships in population data using identity-by-descent and identity-by-state. PloS Genet. 2011; 7(9): e1002287. doi: ARTN e1002287. 10.1371/journal.pgen.1002287 21966277 PMC3178600

[10] Browning SR, Thompson EA. Detecting rare variant associations by identity-by-descent mapping in case-control studies. Genetics. 2012; 190(4):1521–1531. 10.1534/genetics.111.136937 22267498 PMC3316661

[11] Westerlind H, Imrell K, Ramanujam R, Myhr KM, Celius EG, Harbo HF, et al Identity-by-descent mapping in a Scandinavian multiple sclerosis cohort. Eur J Hum Genet. 2015; 23(5):688–692. 10.1038/ejhg.2014.155 25159868 PMC4402631

[12] Beyene Y, Semagn K, Mugo S, Tarekegne A, Babu R, Meisel B, et al Genetic gains in grain yield through genomic selection in eight bi-parental maize populations under drought stress. Crop Sci. 2015; 55(1):154–163.

[13] Liu H, Wang X, Warburton ML, Wen W, Jin M, Deng M, et al Genomic, transcriptomic, and phenomic variation reveals the complex adaptation of modern maize breeding. Mol Plant. 2015; 8(6):871–884. 10.1016/j.molp.2015.01.016 25620769

[14] Troyer AF, Hendrickson LG. Background and importance of ‘Minnesota 13’ corn. Crop Science. 2007; 47(3):905–914.

[15] Li H, Peng Z, Yang X, Wang W, Fu J, Wang J, et al Genome-wide association study dissects the genetic architecture of oil biosynthesis in maize kernels. Nature Genetics. 2013; 45(1):43–50. 10.1038/ng.2484 23242369

[16] Peng B, Li YX, Wang Y, Liu C, Liu ZZ, Tan W, et al QTL analysis for yield components and kernel-related traits in maize across multi-environments. Theor Appl Genet. 2011; 122(7):1305–1320. 10.1007/s00122-011-1532-9 21286680

[17] Schaefer CM, Bernardo R. Population structure and single nucleotide polymorphism diversity of historical Minnesota maize inbreds. Crop Science. 2013; 53(4):1529–1536.

[18] Lu YL, Yan JB, Guimaraes CT, Taba S, Hao ZF, Gao SB, et al Molecular characterization of global maize breeding germplasm based on genome-wide single nucleotide polymorphisms. Theor Appl Genet. 2009;120(1):93–115. 10.1007/s00122-009-1162-7 19823800

[19] Wang R, Yu Y, Zhao J, Shi Y, Song Y, Wang T, et al Population structure and linkage disequilibrium of a mini core set of maize inbred lines in China. Theor Appl Genet. 2008; 117(7):1141–1153. 10.1007/s00122-008-0852-x 18696041

[20] Yan J, Shah T, Warburton ML, Buckler ES, McMullen MD, Crouch J. Genetic characterization and linkage disequilibrium estimation of a global maize collection using SNP markers. PloS One. 2009; 4(12):e8451 10.1371/journal.pone.0008451 20041112 PMC2795174

[21] Yang XH, Gao SB, Xu ST, Zhang ZX, Prasanna BM, Li L, et al Characterization of a global germplasm collection and its potential utilization for analysis of complex quantitative traits in maize. Mol Breeding. 2011; 28(4):511–526.

[22] Hufford MB, Xu X, van Heerwaarden J, Pyhajarvi T, Chia JM, Cartwright RA, et al Comparative population genomics of maize domestication and improvement. Nature Genetics. 2012; 44(7):808–U118. 10.1038/ng.2309 22660546 PMC5531767

[23] Yu YT, Li GK, Yang ZL, Hu JG, Zheng JR, Qi XT. Identification of a major quantitative trait locus for ear size induced by space flight in sweet corn. Genet Mol Res. 2014; 13(2):3069–3078. 10.4238/2014.April.17.3 24782164

[24] Zambrano JL, Jones MW, Francis DM, Tomas A, Redinbaugh MG. Quantitative trait loci for resistance to maize rayado fino virus. Mol Breeding. 2014; 34(3):989–996.

[25] Yang N, Lu Y, Yang X, Huang J, Zhou Y, Ali F, et al Genome wide association studies using a new nonparametric model reveal the genetic architecture of 17 agronomic traits in an enlarged maize association panel. Plos Genet. 2014; 10(9):e1004573 10.1371/journal.pgen.1004573 25211220 PMC4161304

[26] Teng F, Zhai L, Liu R, Bai W, Wang L, Huo D, et al *ZmGA3ox2*, a candidate gene for a major QTL, *qPH3*.*1*, for plant height in maize. Plant J. 2013; 73(3):405–416. 10.1111/tpj.12038 23020630

[27] Lai JS, Li RQ, Xu X, Jin WW, Xu ML, Zhao HN, et al Genome-wide patterns of genetic variation among elite maize inbred lines. Nature Genetics. 2010; 42(11):1027–U158. 10.1038/ng.684 20972441

[28] Jiao Y, Zhao H, Ren L, Song W, Zeng B, Guo J, et al Genome-wide genetic changes during modern breeding of maize. Nature Genetics. 2012; 44(7):812–815. 10.1038/ng.2312 22660547

[29] Wu X, Li Y, Li X, Li C, Shi Y, Song Y, et al Analysis of genetic differentiation and genomic variation to reveal potential regions of importance during maize improvement. BMC Plant Biol. 2015; 15: e256. 10.1186/s12870-015-0646-7 PMC4620006 26496865

[30] Saghai-Maroof MA, Soliman KM, Jorgensen RA, Allard RW. Ribosomal DNA spacer-length polymorphisms in barley: mendelian inheritance, chromosomal location, and population dynamics. Proceedings of the National Academy of Sciences of the United States of America. 1984; 81(24):8014–8018. 6096873 10.1073/pnas.81.24.8014 PMC392284

[31] Flint-Garcia SA, Thuillet AC, Yu JM, Pressoir G, Romero SM, Mitchell SE, et al Maize association population: a high-resolution platform for quantitative trait locus dissection. Plant Journal. 2005; 44(6):1054–1064. 10.1111/j.1365-313X.2005.02591.x 16359397

[32] Pritchard JK, Stephens M, Donnelly P. Inference of population structure using multilocus genotype data. Genetics. 2000; 155(2):945–959. 10835412 10.1093/genetics/155.2.945 PMC1461096

[33] Legesse BW, Myburg AA, Pixley KV, Botha AM. Genetic diversity of African maize inbred lines revealed by SSR markers. Hereditas. 2007; 144(1):10–17. 10.1111/j.2006.0018-0661.01921.x 17567435

[34] Kim EJ, Sa KJ, Park KC, Lee JK. Study of genetic diversity and relationships among accessions of foxtail millet [*Setaria italica* (L.) P. Beauv.] in Korea, China, and Pakistan using SSR markers. Genes Genom. 2012; 34(5):529–538.

[35] Patterson N, Price AL, Reich D. Population structure and eigenanalysis. PloS Genet. 2006; 2(12):2074–2093. 10.1371/journal.pgen.0020190 PMC1713260 17194218

[36] Bradbury PJ, Zhang Z, Kroon DE, Casstevens TM, Ramdoss Y, Buckler ES. TASSEL: software for association mapping of complex traits in diverse samples. Bioinformatics. 2007; 23(19):2633–2635. 10.1093/bioinformatics/btm308 17586829

[37] Liu KJ, Muse SV. PowerMarker: an integrated analysis environment for genetic marker analysis. Bioinformatics. 2005; 21(9):2128–2129. 10.1093/bioinformatics/bti282 15705655

[38] Brown PJ, Upadyayula N, Mahone GS, Tian F, Bradbury PJ, Myles S, et al Distinct genetic architectures for male and female inflorescence traits of maize. PloS Genet. 2011; 7(11):e1002383 10.1371/journal.pgen.1002383 22125498 PMC3219606

[39] Lipka AE, Tian F, Wang QS, Peiffer J, Li M, Bradbury PJ, et al GAPIT: genome association and prediction integrated tool. Bioinformatics. 2012; 28(18):2397–2399. 10.1093/bioinformatics/bts444 22796960

[40] Yu JM, Pressoir G, Briggs WH, Bi IV, Yamasaki M, Doebley JF, et al A unified mixed-model method for association mapping that accounts for multiple levels of relatedness. Nature Genetics. 2006; 38(2):203–208. 10.1038/ng1702 16380716

[41] Schnable PS, Ware D, Fulton RS, Stein JC, Wei FS, Pasternak S, et al The B73 Maize Genome: Complexity, Diversity, and Dynamics. Science. 2009; 326(5956):1112–1115. 10.1126/science.1178534 19965430

[42] Schnable JC, Freeling M, Lyons E. Genome-Wide analysis of syntenic gene deletion in the grasses. Genome Biol Evol. 2012; 4(3):265–277. 10.1093/gbe/evs009 22275519 PMC3318446

[43] Shannon P, Markiel A, Ozier O, Baliga NS, Wang JT, Ramage D, et al Cytoscape: A software environment for integrated models of biomolecular interaction networks. Genome Res. 2003; 13(11):2498–2504. 10.1101/gr.1239303 14597658 PMC403769

[44] Ducrocq S, Madur D, Veyrieras JB, Camus-Kulandaivelu L, Kloiber-Maitz M, Presterl T, et al Key impact of *Vgt1* on flowering time adaptation in maize: Evidence from association mapping and ecogeographical information. Genetics. 2008; 178(4):2433–2437. 10.1534/genetics.107.084830 18430961 PMC2323828

[45] Thornsberry JM, Goodman MM, Doebley J, Kresovich S, Nielsen D, Buckler ES. *Dwarf8* polymorphisms associate with variation in flowering time. Nature Genetics. 2001; 28(3):286–289. 10.1038/90135 11431702

[46] Bouchet S, Servin B, Bertin P, Madur D, Combes V, Dumas F, et al Adaptation of maize to temperate climates: mid-density genome-wide association genetics and diversity patterns reveal key genomic regions, with a major contribution of the *Vgt2* (*ZCN8*) locus. PLoS One. 2013; 8(8):e71377 10.1371/journal.pone.0071377 24023610 PMC3758321

[47] Hung HY, Shannon LM, Tian F, Bradbury PJ, Chen C, Flint-Garcia SA, et al *ZmCCT* and the genetic basis of day-length adaptation underlying the postdomestication spread of maize. Proceedings of the National Academy of Sciences of the United States of America. 2012; 109(28):E1913–E1921. 10.1073/pnas.1203189109 22711828 PMC3396540

[48] Vega SH, Sauer M, Orkwiszewski JA, Poethig RS. The early phase change gene in maize. Plant Cell. 2002; 14(1):133–147. 10.1105/tpc.010406 11826304 PMC150556

[49] Wang TY, Ma XL, Li Y, Bai DP, Liu C, Liu ZZ, et al Changes in yield and yield Components of yingle-cross maize hybrids released in China between 1964 and 2001. Crop Sci. 2011; 51(2):512–525.

[50] Passarinho PA, Van Hengel AJ, Fransz PF, de Vries SC. Expression pattern of the *Arabidopsis thaliana AtEP3*/*AtchitIV* endochitinase gene. Planta. 2001; 212(4):556–567. 10.1007/s004250000464 11525512

