# Supplementary figures and images for: Exploring Identity-By-Descent Segments and Putative Functions Using Different Foundation Parents in Maize
Source: PLoS One. 2016 Dec 20;11(12):e0168374. doi: 10.1371/journal.pone.0168374 (PMC5172581; doi:10.1371/journal.pone.0168374)

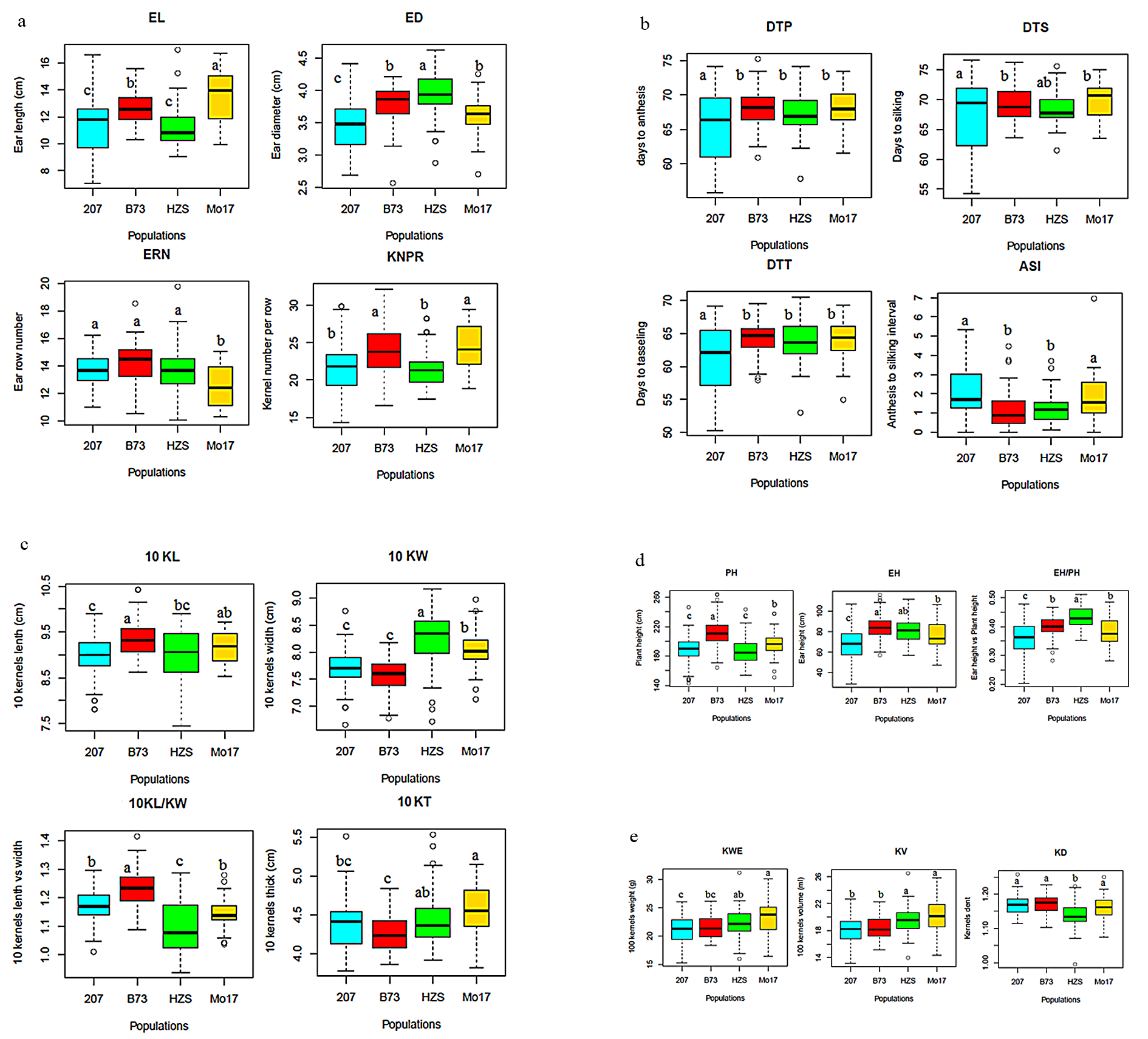

Supplement: S1 Fig — The letter on each box shows multiple testing, and a significant difference at 0.05 between two groups is indicated with different letters. “a” shows ear-related traits, including ear length (EL), ear diameter (ED), ear row number (ER), and kernel number per row (KNPR). “b” shows flowering time-related traits, including DTP, DTS, DTT, and ASI. “c” shows kernel shape-related traits, including 10-kernel length (10KL), 10-kernel width (10KW), 10KL/10KW, and 10-kernel thickness (10KT). “d” shows plant architecture-related traits, including plant height (PH), ear height (EH), and EH/PH. “e” shows kernel weight-related traits, including 100-kernel weight (KWE), 100-kernel volume (KV), and kernel density (KD). (TIF) [file pone.0168374.s001.tif]

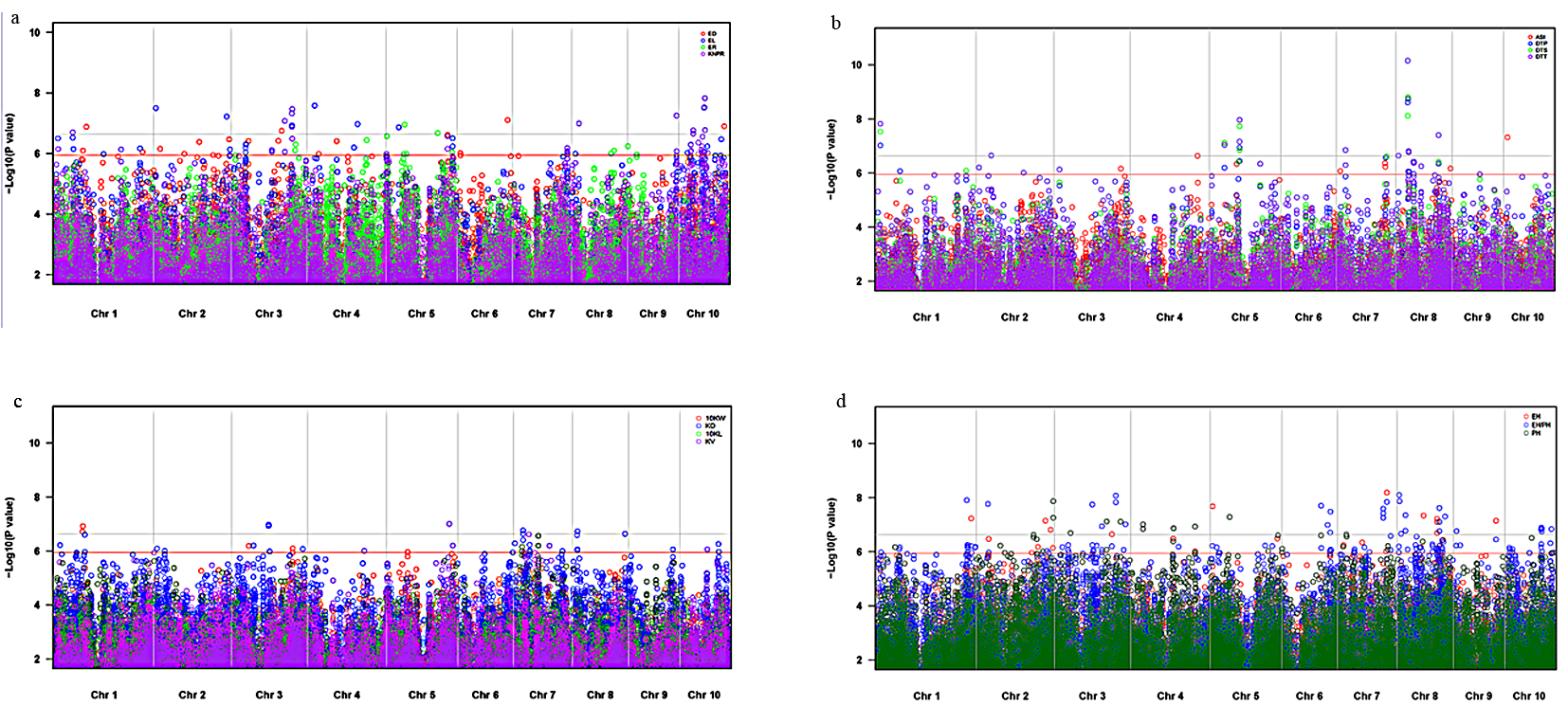

Supplement: S2 Fig — Red and gray lines are defined as 0.05 and 0.01 divided by the SNP number of 43,252, respectively. “a” shows ear-related traits, including ear length (EL), ear diameter (ED), ear row number (ER), and kernel number per row (KNPR). “b” shows flowering time-related traits, including DTP, DTS, DTT, and ASI. “c” shows yield-related traits, including 10-kernel length (10KL), 10-kernel width (10KW), kernel density (KD) and 100-kernel volume (KV). (TIF) [file pone.0168374.s002.tif]
